# Exploring the interplay between posttraumatic stress disorder, gut microbiota, and inflammatory biomarkers: a comprehensive meta-analysis

**DOI:** 10.3389/fimmu.2024.1349883

**Published:** 2024-02-12

**Authors:** Pavlo Petakh, Valentyn Oksenych, Iryna Kamyshna, Iryna Boisak, Katerina Lyubomirskaya, Oleksandr Kamyshnyi

**Affiliations:** ^1^ Department of Biochemistry and Pharmacology, Uzhhorod National University, Uzhhorod, Ukraine; ^2^ Department of Microbiology, Virology, and Immunology, I. Horbachevsky Ternopil National Medical University, Ternopil, Ukraine; ^3^ Broegelmann Research Laboratory, Department of Clinical Science, University of Bergen, Bergen, Norway; ^4^ Department of Medical Rehabilitation, I. Horbachevsky Ternopil National Medical University, Ternopil, Ukraine; ^5^ Department of Childhood Diseases, Uzhhorod National University, Uzhhorod, Ukraine; ^6^ Department of Obstetrics and Gynecology, Zaporizhzhia State Medical and Pharmaceuticals University, Zaporizhzhia, Ukraine

**Keywords:** gut microbiome, stress, post-traumatic stress disorder, inflammation, IL-6, *Lachnospiraceae*

## Abstract

**Introduction:**

Posttraumatic stress disorder (PTSD) is the most common mental health disorder to develop following exposure to trauma. Studies have reported conflicting results regarding changes in immune biomarkers and alterations in the abundance of bacterial taxa and microbial diversity in patients with PTSD.

**Aim:**

The purpose of this meta-analysis is to summarize existing studies examining gut microbiota characteristics and changes in immune biomarkers in patients with PTSD.

**Methods:**

Relevant studies were systematically searched in PubMed, Scopus, and Embase, published in English between January 1, 1960, and December 1, 2023. The outcomes included changes in abundance and diversity in gut microbiota (gut microbiota part) and changes in immune biomarkers (immune part).

**Results:**

The meta-analysis included a total of 15 studies, with 9 focusing on changes in inflammatory biomarkers and 6 focusing on changes in gut microbiota composition in patients with PTSD. No differences were observed between groups for all inflammatory biomarkers (P≥0.05). Two of the six studies found that people with PTSD had less alpha diversity. However, the overall Standardized Mean Difference (SMD) for the Shannon Diversity Index was not significant (SMD 0.27, 95% CI -0.62–0.609, p = 0.110). Regarding changes in abundance, in two of the studies, a significant decrease in Lachnospiraceae bacteria was observed.

**Conclusion:**

This meta-analysis provides a comprehensive overview of gut microbiota characteristics in PTSD, suggesting potential associations with immune dysregulation. Future research should address study limitations, explore causal relationships, and consider additional factors influencing immune function in individuals with PTSD.

**Systematic review registration:**

https://www.crd.york.ac.uk, identifier CRD42023476590.

## 1 Introduction

Posttraumatic stress disorder (PTSD) is a debilitating mental health disorder that can develop following exposure to trauma (1). It is characterized by distinct symptoms, including re-experiencing traumatic events, avoiding reminders of the trauma, heightened arousal, and negative changes in cognition and mood (2). PTSD is the most common mental health disorder that arises after trauma and has significant impacts on individuals’ well-being and daily functioning (3).

Currently, the world is witnessing new and escalating military conflicts, with the Russian-Ukrainian conflict being one of the largest and most significant. Since February 2022, this conflict has resulted in numerous casualties among both civilian and military populations, as well as massive displacement of people (4–7). The psychological toll of such experiences, including stress and the risk of developing PTSD, is a pressing concern that needs addressing (8, 9).

Recent research has shed light on the intricate relationship between the gut microbiota and mental health (10). The gut microbiota, comprised of trillions of microorganisms residing in the digestive system, has been found to play a crucial role in influencing brain function through the ‘microbiota-gut-brain axis (11, 12). Disruptions in the composition and functioning of the gut microbiota have been associated with various psychiatric disorders (13).

Intestinal bacteria are also capable of modulating the immune response, both individually and as consortia, as well as through their metabolites (14–21). These metabolites can exert direct effects on immune cells and can also interact with the gut-brain axis, thereby further influencing brain function (22). Additionally, studies have shown that specific strains of gut bacteria can produce neurotransmitters and other molecules that can directly impact brain activity and behavior (23). Understanding the characteristics of the gut microbiota in individuals with PTSD can provide valuable insights into the underlying mechanisms and potentially open up new avenues for therapeutic interventions.

The purpose of this meta-analysis is to review and summarize existing studies that have examined the gut microbiota characteristics in patients with PTSD. Additionally, the review will compare the levels of inflammatory biomarkers in patients to understand the potential relationship between the gut microbiota, PTSD, and immune biomarkers.

Understanding the characteristics of the gut microbiota in individuals with PTSD can have several implications. Firstly, it can help identify biomarkers or specific microbial signatures associated with the disorder, which can aid in the diagnosis and early detection of PTSD. Secondly, it can inform the development of targeted therapeutic interventions that focus on modulating the gut microbiota to improve mental health outcomes in patients with PTSD. Thirdly, it can contribute to a deeper understanding of the underlying mechanisms of PTSD and potentially uncover novel pathways for intervention. By elucidating the complex interplay between the gut microbiota, PTSD, and immune biomarkers, this research has the potential to revolutionize our approach to the prevention and treatment of PTSD.

## 2 Methods

### 2.1 Inclusion and exclusion criteria

The eligibility criteria were established to include studies that investigated the gut microbiota composition in individuals diagnosed with PTSD. The inclusion and exclusion criteria were carefully defined to ensure the selection of relevant studies.

### 2.2 Search strategy

The search strategy followed the Preferred Reporting Items for Systematic Reviews and Meta-Analyses (PRISMA) guidelines (24). We conducted a comprehensive search across multiple databases, including PubMed, Scopus, and Embase, to identify relevant studies published in English between January 1, 1960, and December 1, 2023. The following keywords were used: (“Inflammation” OR “Immune Activation” OR “Interleukin” OR “Cytokine” OR “Interferon” OR “Lymphocyte” OR “Macrophage” OR “Tumor Necrosis Factor-alpha” OR “C-Reactive Protein” OR “IL-1” OR “IL-2” OR “IL-4” OR “IL-6” OR “IL-8” OR “IL-10” OR “Interferon” OR “IFN” OR “TNF”) AND “Posttraumatic Stress Disorder” OR “PTSD”. For searching articles related to gut microbiota, the following keywords were used: “Gut Microbiota” OR “Intestinal Microbiota” OR “Microbial Composition” OR “Microbiome” OR “Bacterial Diversity” OR “Microbial Dysbiosis” OR “Bacterial Metabolites” OR “Microbial Modulation” OR “Microorganism Influence”) AND (“Posttraumatic Stress Disorder” OR “PTSD” OR “Trauma-Induced Psychopathology” OR “Psychiatric Sequelae of Trauma” OR “Stress-Related Disorders.”

### 2.3 Data extraction and data analysis

Two independent reviewers screened the titles and abstracts of all identified records to assess their eligibility for inclusion in the meta-analysis. The reviewers resolved any disagreements through discussion and consensus. The same reviewers retrieved and reviewed full-text articles of potentially eligible studies to determine final inclusion. Two reviewers performed data extraction independently and resolved any discrepancies through consensus. If other statistics were reported instead of mean and SD, we requested the data from the corresponding author via email. If this approach failed, we used the estimation method to calculate SD according to the Cochrane Handbook for Systematic Reviews of Interventions (25). We used Comprehensive Meta-Analysis V3 for all analyses (24).

If the number of studies that included a specific marker was equal to or exceeded three, we performed meta-analyses on individual immune markers. These main analyses were based on random-effects models. If heterogeneity is significant, a random-effects model is chosen for meta-analysis as it assumes that the underlying true effects vary from one trial to another (26). We used standardized mean difference (SMD) and 95% confidence intervals (CI) to assess the effect size. The significance level was defined as P< 0.05. An effect size of 0.2 or less was considered a low effect, 0.5 or more was a large effect.

We obtained publication details, participant demographic and clinical characteristics, and methodological information from systematic reviews and original studies. The main outcomes we focused on were community-level measures of gut microbiota composition, specifically alpha and beta diversity. We also examined taxonomic findings at the phylum, family, and genus levels, specifically looking at relative abundance. Alpha diversity offers a concise overview of the microbial community within individual samples and allows for comparisons between groups to assess the impact of a specific factor (in this instance, psychiatric diagnosis) on the abundance (number of species) and uniformity (representation of each species) within the sample. Beta diversity quantifies the degree of dissimilarity between samples, evaluating the similarity of communities in relation to the other analyzed samples.

### 2.4 Heterogeneity

We assessed heterogeneity using Q-tests and quantified the proportion of total variability due to heterogeneity with the I^2^ statistic. An I^2^value <50% was considered low heterogeneity, I^2^ ≥ 50% but <75% considered medium heterogeneity, and I2 ≥ 75% considered high heterogeneity. The presence of significant heterogeneity suggests that the characteristics of studies are divergent.

### 2.5 Quality assessment

Two researchers used the Newcastle-Ottawa Quality Assessment Scale to assess the quality of the literature, and a third reviewer helped resolve differences when necessary (24). The NOS included three criteria: selectivity, comparability, and exposure. Each study could receive up to nine stars. A study with a score of ≥7 was considered of good quality, with a score of 5−6 of average quality, and a score of 0−4 was of poor quality. To visualize the risk of bias, we employed the Robvis tool (27).

## 3 Results

**Figure 1:**
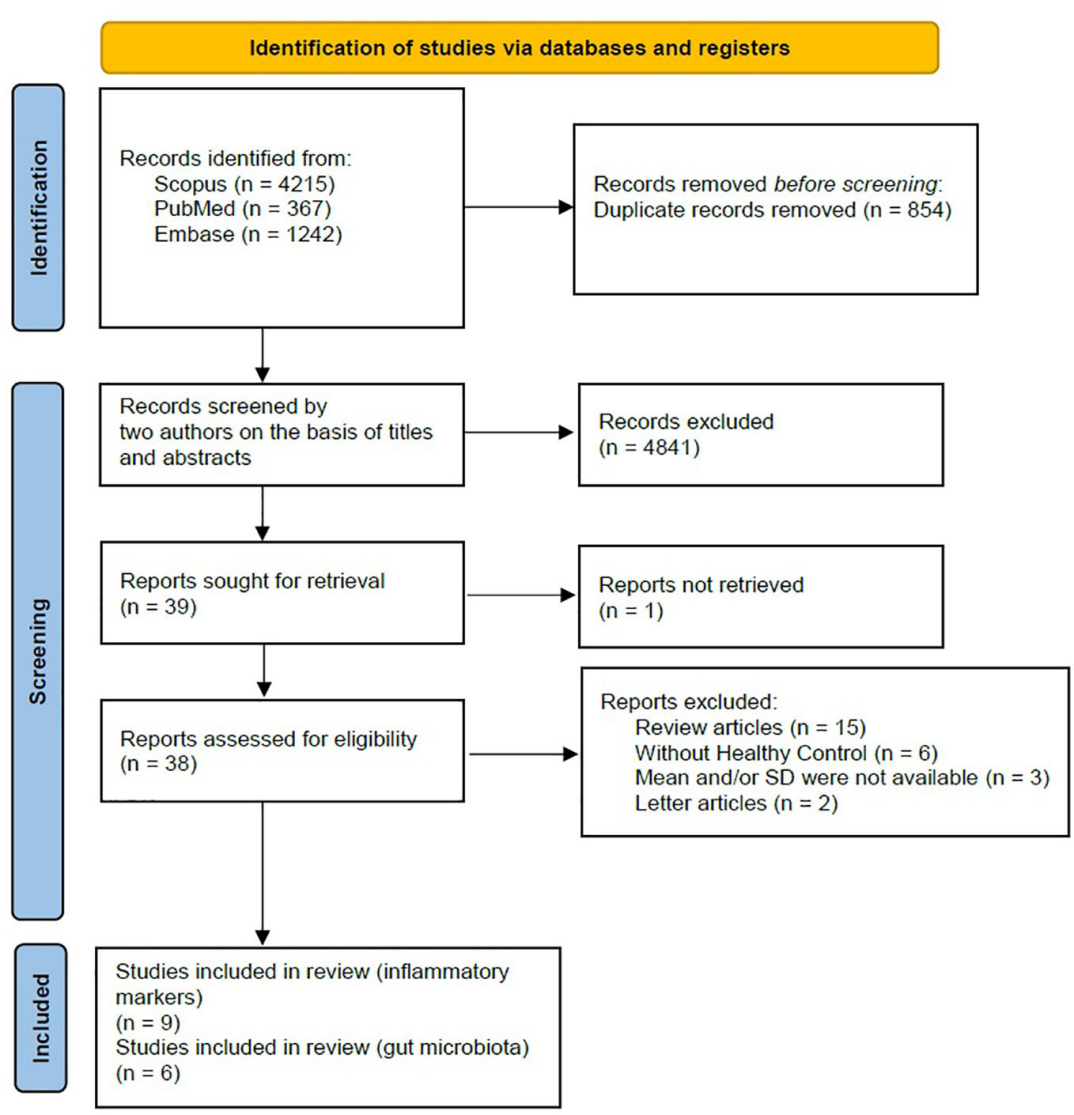
PRISMA 2020 flow diagram.

**Figure 2:**
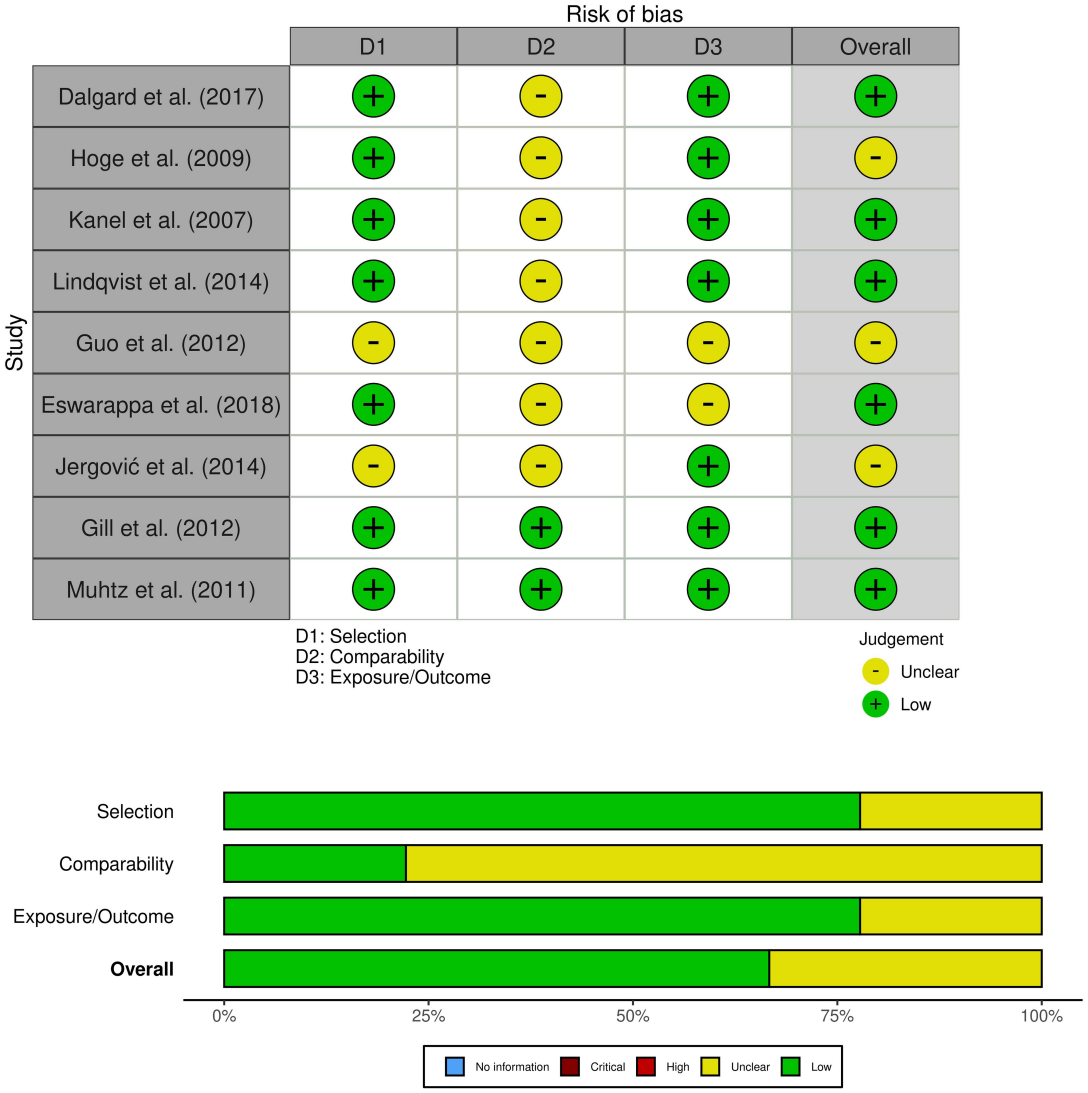
Risk of bias (studies of inflammation biomarkers).

**Figure 3:**
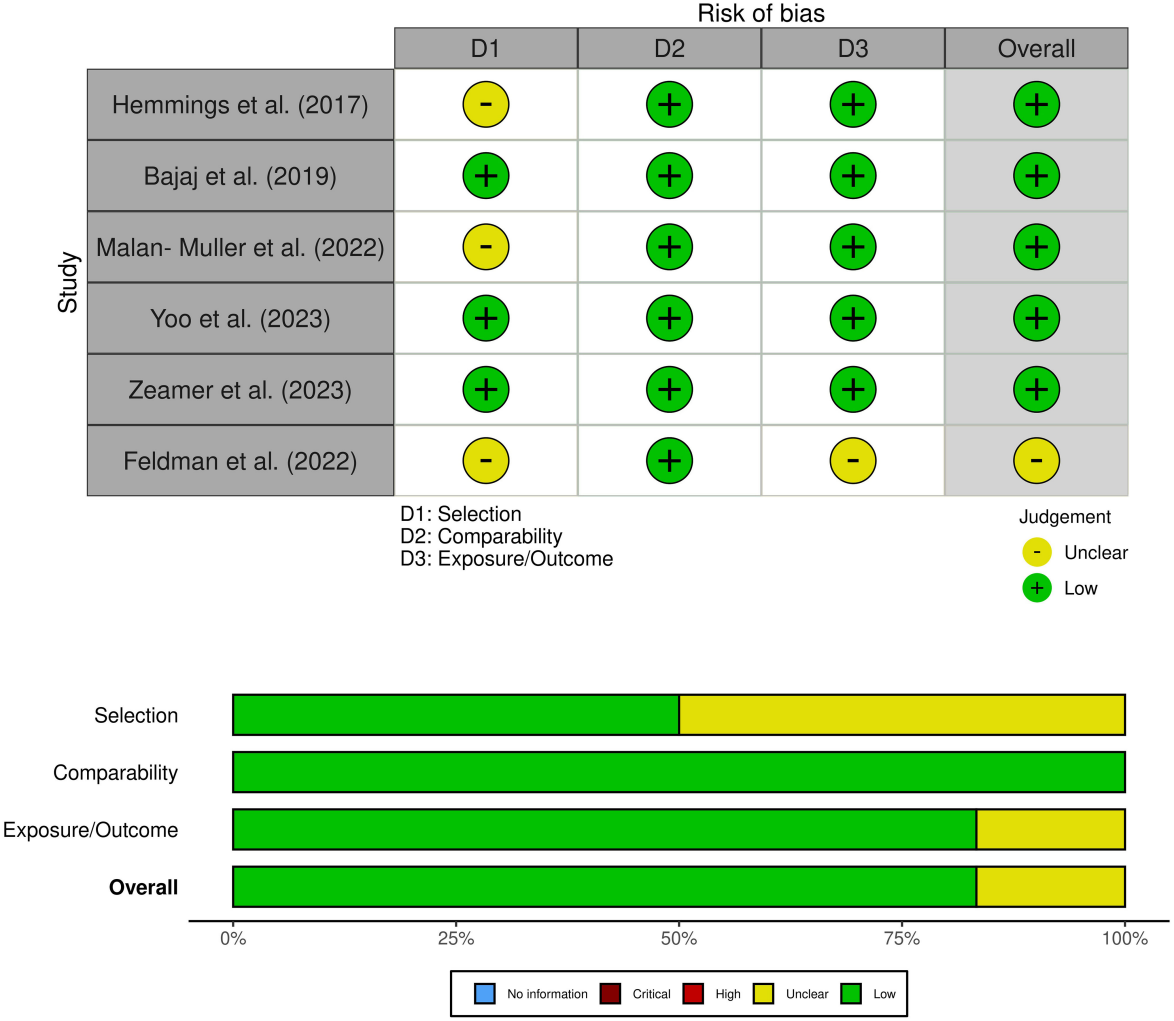
Risk of bias (studies of gut microbiota composition).

A comprehensive search was conducted on a total of 5824 articles, out of which 854 were eliminated from consideration due to duplicate findings. Additionally, a significant number of individuals were excluded based on the evaluation of their title or abstract (n = 4841). After analyzing the remaining 39 articles in their entirety, 26 were excluded due to various reasons (refer to **Figure 1**). Our meta-analysis included a total of 15 studies, with 9 focusing on changes in inflammatory biomarkers and 6 focusing on changes in gut microbiota composition in patients with PTSD. **Table 1** describes the characteristics of the included studies, outlining essential details such as sex, sample size, inflammatory markers included in the meta-analysis, and diagnostic criteria for PTSD. In total, among the 9 articles examining inflammatory biomarkers in individuals with PTSD, 401 had a confirmed diagnosis of PTSD, and an additional 642 served as comparison controls. The total number of participants in the gut microbiota sub-part was 489. Risk of bias was assessed using the Newcastle-Ottawa Quality Assessment Scale, as illustrated in **Figures 2** and **3**.

**Table 1:** Characteristics of studies included in the meta-analysis.

1. Inflammatory markers					
Study (year)	Inflammatory markers included in meta-analysis	Number participants	Gender (male %) (PTSD/HC)	Sample type	PTSD Diagnosis
Dalgard et al. (2017) (28)	IL-1β, IL-2, IL-6,IL-8, IL-10, TNF-α	PTSD (n=16); HC (n=11)	31.2/45.4	Plasma	DSM-IV
Hoge et al. (2009) (29)	IL-1β*, IL-2,* IL-4*, IL-6*, IL-8*, IL-10*, TNF-α*	PTSD (n=28); HC (n=48)	50/50	Plasma	DSM-IV
Kanel et al. (2007) (30)	IL-1β, IL-4, IL-6, IL-10, CRP	PTSD (n=14); HC (n=14)	64/64	Plasma	DSM-IV, CAPS
Lindqvist et al. (2014) (31)	IL-1β, IL-6, IL-10, CRP, TNF-α*	PTSD (n=51); HC (n=51)	100/100	Serum	DSM-IV, CAPS
Guo et al. (2012) (32)	IL-2, IL-4, IL-6, IL-8, IL-10, TNF-α	PTSD (n=50); HC (n=50)	44/50	Serum	DSM-IV
Eswarappa et al. (2018) (33)	IL-6, CRP*	Chronic PTSD (n=170); HC (n=396)	91.2/97	Plasma	DSM IV
Jergović et al. (2014) (34)	CRP	PTSD (n=21); HC (n=23)	100/100	Serum	ICD-10
Gill et al. (2012) (35)	IL-6*, CRP*	PTSD (n=26); HC (n=24)	0/0	Plasma	DSM IV
Muhtz et al. (2011) (36)	CRP	PTSD (n=25); HC (n=25)	36/36	Plasma	PDS/BDI
2. Gut microbiota					
Study (year)	Number participants	Sample type	Gender (male %) (PTSD/HC)	Method of analysis	PTSD Diagnosis
Hemmings et al. (2017) (37)	PTSD (n=18); Trauma-exposed control (n=12)	Stool samples	22.2/41.7	16S rRNA sequencing	CAPS-5
Bajaj et al. (2019) (38)	PTSD (n = 29); Control (n=64);	Stool samples	100/100	16S rRNA sequencing	DSM-V
Malan- Muller et al. (2022) (39)	PTSD (n = 79); Trauma-exposed control (n = 58);	Stool samples	20.26/18.97	16S rRNA sequencing	CAPS-5
Yoo et al. (2023) (40)	Firefighters (n = 15); Control (n = 15);	Stool samples	100/100	16S rRNA sequencing	PCL-C
Zeamer et al. (2023) (41)	Microbiome Sub-study (n = 51)	Stool samples	49	16S rRNA sequencing	DSM-V
Feldman et al. (2022) (42)	Mother-child dyads from Sderot, Israel (n = 148)	Stool samples	47.6	16S rRNA sequencing	DC:0-3R

**Figure 4:**
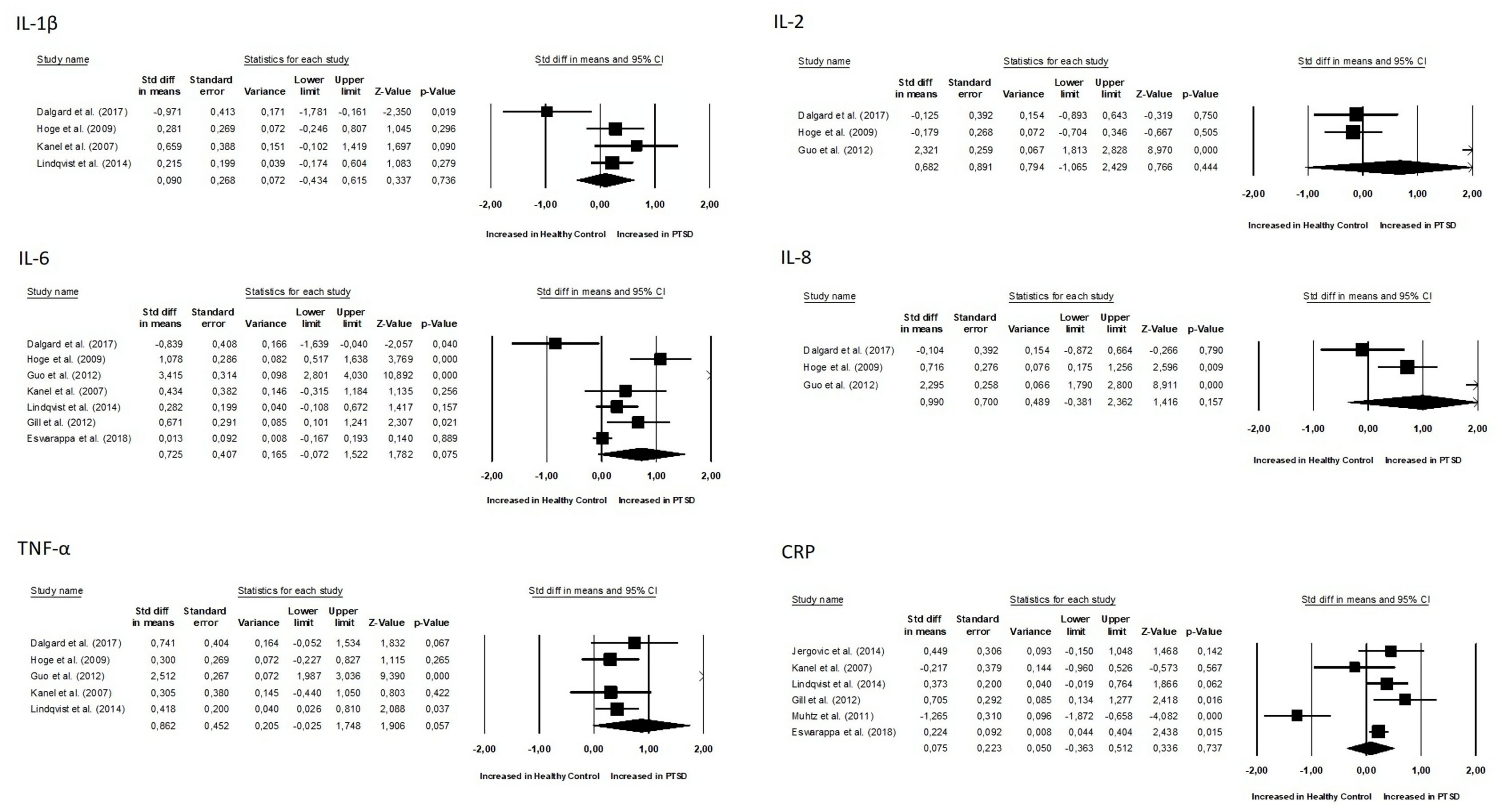
Meta-analyses of pro-inflammatory biomarkers (IL-1β, IL-2, IL-6, IL-8, TNF-α, CRP).

**Figure 5:**
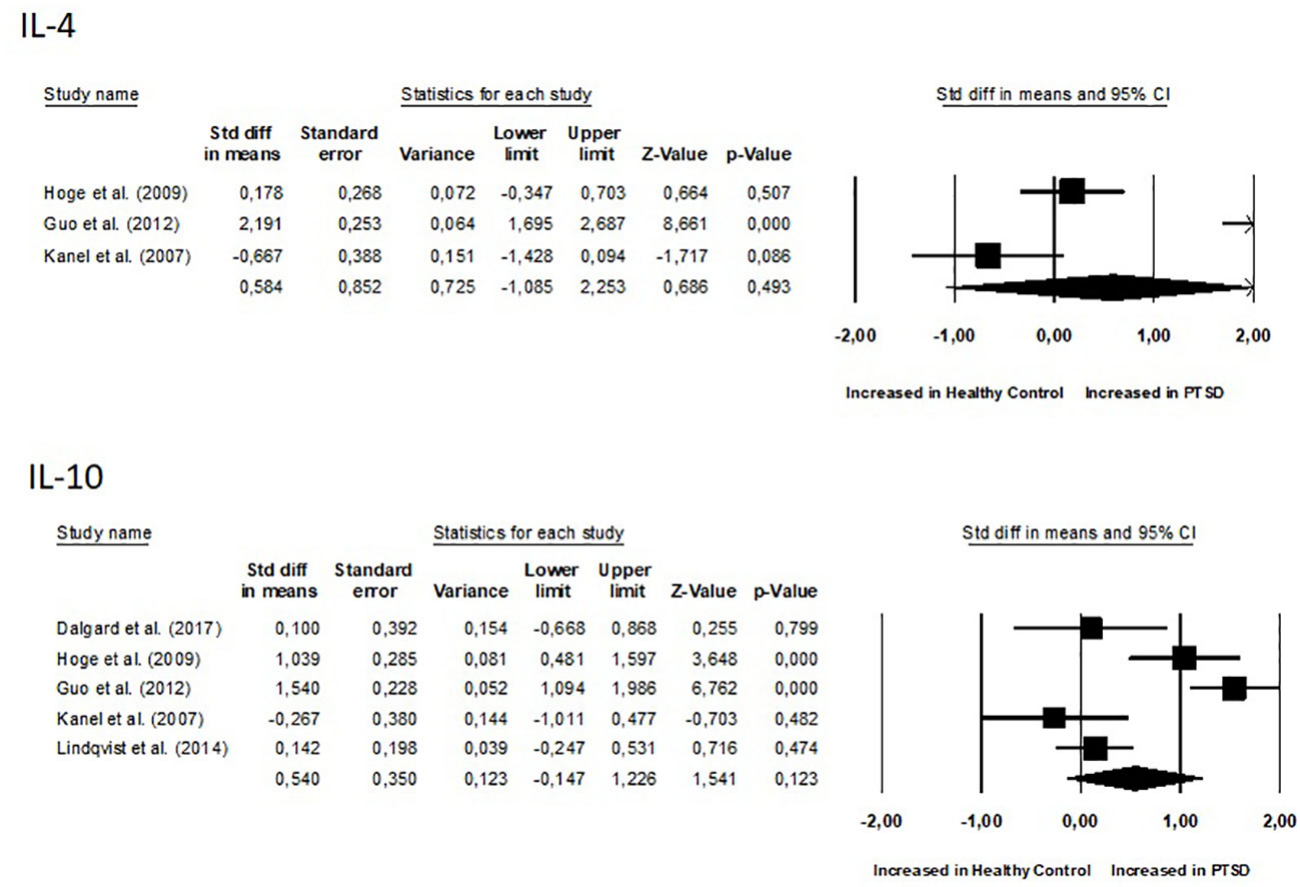
Meta-analyses of anti-inflammatory biomarkers (IL-4 and IL-10).

No differences were observed between groups for all inflammatory biomarkers (P≥0.05), as depicted in **Figures 4** and **5**. TNF-α (SMD 0.86, 95% CI -0.02 – 1.74, p = 0.057) and IL-6 (SMD 0.72, 95% CI -0.07 – 1.52, p = 0.075) had slightly higher p-values. Additionally, for all inflammatory markers, study heterogeneity was reported to be high (I² > 75%), except for the study on interleukin-1β, where heterogeneity was 68% (medium).

Additionally, it was crucial to determine whether there were correlations between inflammation biomarkers and the course’s severity. Out of the nine studies, only three conducted a correlational study between inflammatory markers and PTSD severity. Notably, one study found that PTSD patients had significantly higher pro-inflammatory scores compared to combat-exposed subjects without PTSD. However, the pro-inflammatory score was not significantly correlated with depressive symptom severity, CAPS total score, or the number of early-life traumas (31). In another study, TNF-α positively correlated with the total (frequency and intensity) PTSD symptom cluster of re-experiencing, avoidance, and hyperarousal, as well as with the PTSD total symptom score. Controlling for time since trauma attenuated these associations. IL-1β positively correlated with symptoms of anxiety and depression. IL-4 negatively correlated with total hyperarousal symptoms, systolic blood pressure (30). In the study conducted by Eswarappa et al., the biomarkers white blood cell count (OR = 1.27, 95% CI: 1.10–1.47, p = 0.001), C-reactive protein (OR = 1.20, 95% CI: 1.04–1.39, p = 0.02), and erythrocyte sedimentation rate (ESR) (OR = 1.17, 95% CI: 1.00–1.36, p = 0.05) were identified as significant predictors of poorer courses of PTSD (33). Since there were few data and studies, we did not calculate correlations of effects indexes.

**Figure 6:**
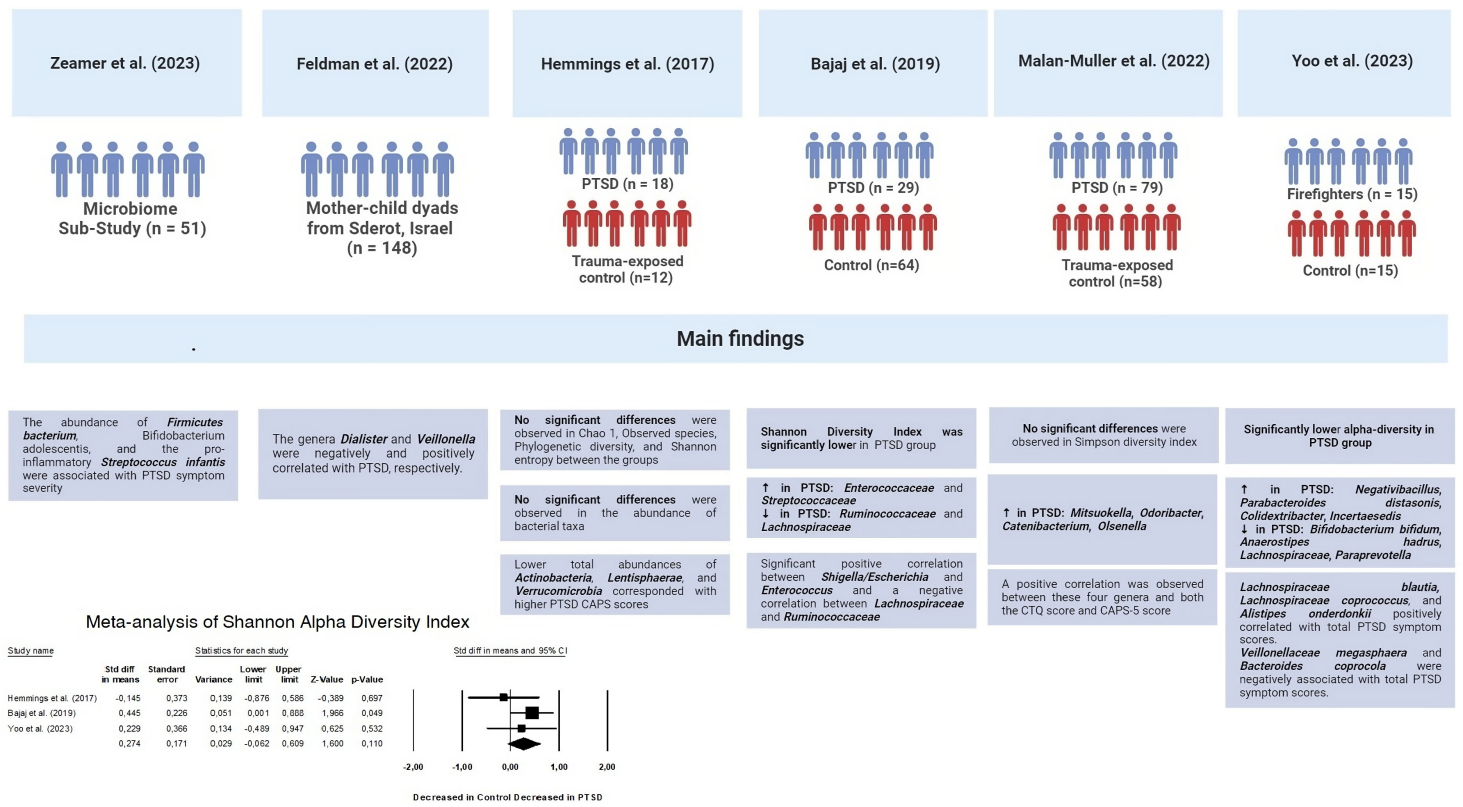
Key findings of gut microbiota composition studies.

The second part of the systematic review involved an analysis of the characteristics of the intestinal microbiota in patients with PTSD (**Figure 6**). Two out of the six studies showed a decrease in alpha diversity in PTSD patients (SMD for Shannon Diversity Index 0.27, 95% CI -0.62 – 0.609, p = 0.110), while the other two studies found no significant difference between the PTSD and control groups. In two of the studies, a significant decrease in *Lachnospiraceae* bacteria was observed. In one of these studies, bacteria of this taxa were positively correlated with PTSD symptoms score, while in the other study, *Lachnospiraceae* were associated with higher cognitive functioning. The remaining results did not show any consistent patterns and were unique to each study.

## 4 Discussion

The findings of this meta-analysis provide valuable insights into the characteristics of the gut microbiota in patients with PTSD. The results suggest that there may be alterations in the diversity and composition of the gut microbiota in individuals with PTSD, as well as potential associations with specific bacterial taxa.

*Lachnospiraceae* are the main SCFA producers of dietary fiber that have anti-inflammatory and modulating effects on the intestinal mucosa, maintaining gut health (10). As we all know, the colon plays a big part in providing energy and trophic factors, as well as controlling T regulatory (Treg) cell colonies (43–45). More and more evidence suggests that SCFAs also have important physiological effects on many organs, including the brain (46–48). Gut microbiota dysbiosis has been linked to behavioral and neurological disorders like autism spectrum disorder (ASD), Alzheimer’s disease (AD), and Parkinson’s disease (PD) in both humans and animals, which supports this idea (49–51). Furthermore, microbiota manipulation and SCFA administration have been proposed as treatment targets for such diseases (52).

While this meta-analysis provides valuable insights into the characteristics of the gut microbiota in patients with PTSD, several limitations should be acknowledged. First, the included studies varied in their sample sizes, diagnostic criteria for PTSD, taking medications (antipsychotics), and taking food. This heterogeneity may have influenced the results and should be considered when interpreting the findings. Second, the cross-sectional nature of the included studies limits our ability to establish causality or determine the temporal relationship between the gut microbiota and PTSD.

One potential mechanism underlying the observed associations between the gut microbiota and PTSD is through the modulation of immune function and inflammation. Disruptions in the gut microbiota composition and functioning have been associated with altered immune responses and increased inflammation, which have been implicated in the pathophysiology of psychiatric disorders, including PTSD (53, 54). Changes in the gut microbiota may lead to dysregulation of the immune system, contributing to the development and maintenance of PTSD symptoms (55). Environmental factors like stress and diet can disturb the gut microbiome, triggering the intestinal epithelium to release pro-inflammatory cytokines, potentially causing intestinal permeability, increased antigen movement, and inflammation (56). Increasing evidence suggests that imbalanced communication within the gut-brain axis plays a role in the development of stress and mood disorders, with observed changes in the gut microbiome in individuals with PTSD (57, 58). Gut microbiome alterations may also mediate the association between early life adversity and symptoms of anxiety in adulthood (59). Along with research showing a strong link between PTSD and inflammatory gastrointestinal conditions like IBD, the data point to a possible role for gut microbiota imbalance in the inflammatory environment linked to PTSD (60).

However, no significant differences were observed in the levels of inflammatory biomarkers between the two groups. There have been two meta-analyses conducted so far. In one meta-analysis by Yang et al. (2020), interleukin-1β, IL-2, IL-6, interferon-γ, TNF-α, C-reactive protein, and white blood cells were higher in PTSD (61). In a meta-analysis, Passos et al. (2015) found that interleukin 6, interleukin 1β, and interferon γ levels were higher in the PTSD group than in healthy controls (62).

PTSD affects the immune system because it overworks the sympathetic nervous system and alters the function of the hypothalamus-pituitary-adrenal (HPA) axis (63). Activating the sympathetic nervous system causes catecholaminergic neurotransmitters, like norepinephrine, to be released, which in turn causes pro-inflammatory cytokines to be released. Catecholamine activates immune responses via the adrenergic-β receptor (64). Previous studies indicated that catecholamine-induced Th1 responses modulated immune cell distribution through the β-adrenergic receptor (65). Interestingly, previous studies found that the concentrations of norepinephrine and the expression of the adrenergic-β2 receptor increased in PTSD patients (66, 67). When the HPA axis was activated, it stopped pro-inflammatory activity by releasing glucocorticoids and stopping the NFκB pathway (68). Previous studies indicated that dysregulation of the HPA axis promoted pro-inflammatory cytokine secretion in PTSD (69). For example, Yehuda et al. found the levels of salivary cortisol were decreased in PTSD, and Klengel et al. found glucocorticoid receptor resistance in PTSD (70, 71). In summary, the results indicated that PTSD patients were in a pro-inflammatory state.

These findings suggest that dysregulation of the immune system may play a role in the development and maintenance of PTSD symptoms, although further research is needed to fully understand the relationship between inflammatory biomarkers and PTSD. Additionally, it is important to consider other factors that may contribute to immune dysregulation in individuals with PTSD, such as lifetime trauma burden, biological sex, genetic background, metabolic conditions. Experiencing trauma and stress throughout one’s life may contribute to inflammation even before the occurrence of a traumatic incident leading to PTSD (72). A cross-diagnostic meta-analysis of trauma exposure showed that people who had a lot of traumatic events in their lives, like being abused as a child, being in a natural disaster, or being in a violent situation, had higher levels of CRP, IL-1β, IL-6, and TNF-α in their blood. To demonstrate the connection between inflammation and the occurrence of traumatic events, researchers have used animal models such as repeated social defeat stress (RSDS). They found that IL-17A released by meningeal T cells in the brain controlled anxious behavior in mice by connecting to IL-17Ra on neurons (62).

Metabolic conditions coexisting with PTSD can potentially intensify the inflammatory environment associated with the disorder (73). People with PTSD are at a heightened risk of developing type 2 diabetes mellitus, metabolic syndrome (MetS), and its various components, such as obesity, insulin resistance, and dyslipidemia (74, 75). This higher rate of comorbidity can be explained by the unhealthy lifestyles that are linked to PTSD, such as irregular sleep patterns, poor nutrition, drug and tobacco use, and lack of physical activity. These lifestyles make inflammation worse (76, 77). The noradrenergic system is turned on in both MetS and PTSD, which starts an innate immune response (73). Similar to PTSD, MetS, and obesity are marked by elevated levels of proinflammatory markers, including CRP, IL-6, and TNF-α (78).

The metabolic findings suggest inflammation, inefficient energy production, and potential mitochondrial dysfunction in individuals with PTSD (77). Mitochondrial dysfunction could result in heightened production of reactive oxygen species (ROS) in peripheral organs and immune cells, contributing to peripheral inflammation. The proposed connection between inflammation, oxidative stress, and metabolism is further emphasized by Kusminski and Scherer, who suggest that mitochondrial dysfunction plays a linking role in these interconnected processes.

## Data availability statement

The raw data supporting the conclusions of this article will be made available by the authors, without undue reservation.

## Author contributions

PP: Conceptualization, Visualization, Writing – original draft. VO: Writing – review & editing. IK: Writing – original draft. IB: Writing – review & editing. KL: Writing – review & editing. OK: Supervision, Visualization, Writing – original draft.
